# Cohort Profile update: The 1993 Pelotas (Brazil) Birth Cohort follow-up visits in adolescence

**DOI:** 10.1093/ije/dyu077

**Published:** 2014-04-11

**Authors:** Helen Gonçalves, Maria CF Assunção, Fernando C Wehrmeister, Isabel O Oliveira, Fernando C Barros, Cesar G Victora, Pedro C Hallal, Ana MB Menezes

**Affiliations:** ^1^Postgraduate Program in Epidemiology, Federal University of Pelotas, Pelotas, Brazil and ^2^Postgraduate Program in Health and Behaviour, Catholic University of Pelotas, Pelotas, Brazil

## Abstract

In this paper we update the profile of the 1993 Pelotas (Brazil) Birth Cohort Study, with emphasis on a shift of priority from maternal and child health research topics to four main categories of outcome variables, collected throughout adolescence: (i) mental health; (ii) body composition; (iii) risk factors for non-communicable diseases (NCDs); (iv) human capital. We were able to trace 81.3% (*n* = 4106) of the original cohort at 18 years of age. For the first time, the 18-years visit took place entirely on the university premises, in a clinic equipped with state-of-the-art equipment for the assessment of body composition. We welcome requests for data analyses from outside scientists. For more information, refer to our website (http://www.epidemio-ufpel.org.projetos_de_pesquisas/estudos/coorte_1993) or e-mail the corresponding author.

**Key Messages**

- It is possible to conduct long-term cohort studies in a middle-income setting and achieve high follow up rates.
- The existence of three birth cohort studies in a 22-year period in the same city allows the study of time trends in health indicators.
- By the age of 18 years, 10% of the participants already had a child, 80% were sexually active, 23% reported having experienced tobacco and 92% alcohol ;and 89% had any paid work during the past year.

## What is the rationale for the new focus?

In the original cohort profile (http://ije.oxfordjournals.org/content/37/4/704.short),^1^ we described how all live-born children in 1993 in the city of Pelotas, Brazil, were followed up until 11 years of age.^1^ In this update we provide information about the follow-up visits at 15 and 18 years of age. The original goals of the 1993 cohort were: (i) to evaluate trends in maternal and child health indicators, by comparing the results with those from the 1982 cohort which had taken place in the same city; (ii) to assess associations between early-life variables and later outcomes, with particular emphasis on the detection of critical windows; and (iii) to improve data quality, using the lessons learned from the 1982 cohort study.^2^

**Figure 1.**
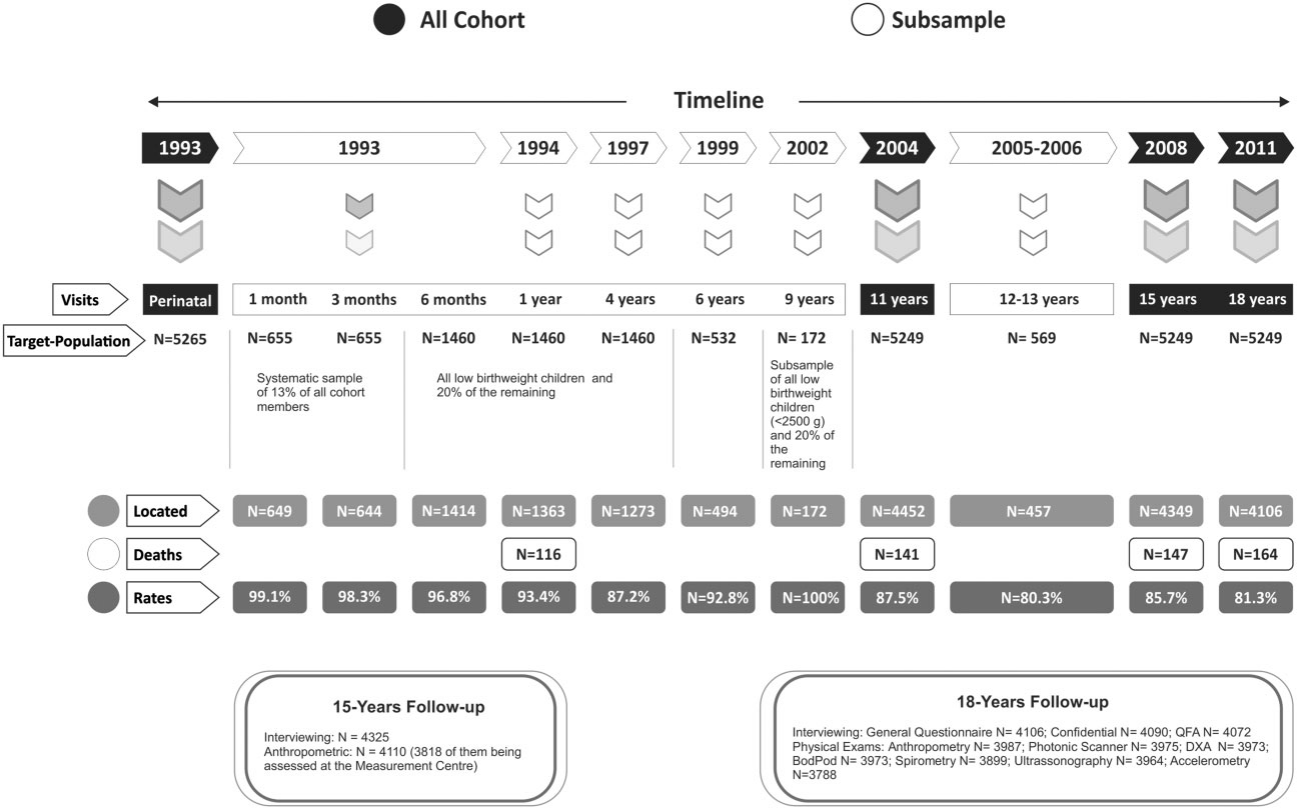
Description of the 1993 Pelotas Birth Cohort. Visits and follow-up rates.

The different follow-up waves of the 1993 cohort are shown in Figure 1. Only subsamples of the participants were sought until they attained the age of 11 years. Visits to the full cohort took place at 11, 15 and 18 years of age. Topic-specific sub-studies were conducted at the ages of 4, 6, 9, 11,^1–2^ 13^3^ and 18 years.

From the mid 2000s onwards, there was a major change in the fieldwork strategy and research priorities. Through a grant obtained from the Wellcome Trust, we were able to use new premises at the university in addition to household visits in order to examine and interview cohort members. The facilities at the university headquarters included state of the art technology of the measurement of body composition, physical activity, lung function and several other predictors of non-communicable diseases (NCDs). This is justified by the new epidemiological profile of the Brazilian population, in which the burden of NCDs is much higher than that of communicable diseases.

## What will be the new areas of research?

The eighth wave of data collection for the 1993 Pelotas birth cohort was carried out in 2008. All participants from the cohort study were sought at the age of 15 years. In 2011, the ninth follow-up wave began, also aiming to locate all participants at the age of 18 years. These waves were focused on four groups of outcome variables: (i) mental health; (ii) body composition; (iii) risk factors for NCDs; and (iv) human capital.

## Who is in the cohort?

### 15-years follow-up

All surviving cohort members were sought in 2008. The first strategy used to trace them was telephone contact by using identification data obtained at the 11-years visit. This led to locating 2234 cohort members. Additional search strategies included: (i) records from government cash transfer programmes; (ii) online phonebooks; (iii) social networks; (iv) state school enrolment records; and (v) private school enrolment records. Additionally, we used previously collected information on relatives and employers of the parents of cohort members to locate those who were still untraced. This active search process led to 1400 more adolescents being found. We also asked participants to contact friends born in 1993 and invite them to visit the research clinic. Simultaneously, advertisements were placed in the local media.

By the time of the 15-years visit, 148 deaths had been identified among the 5249 original cohort members. Out of the 5108 remaining participants, 4325 were interviewed at home. Added to those known to have died, this represents an 85.7% follow-up rate. Of those interviewed, 4110 adolescents had several measurements taken at the university clinic (see below).

The average age of the interviewees was 14.7 years, and 51.0% of them were girls. Selected results from this follow-up visit were published in a special issue of the *Journal of Adolescent Health* and other journals.^4–14^

### 18-years follow-up

Unlike the previous follow-up visits, this wave of the study was planned to take place entirely at the clinic. The workup included interviews and several measurements (see below). In August 2009, approximately 2 years prior to the follow-up, a team started the process of updating addresses, contact persons and phone numbers. Boyss had to enlist in the army, and a study team was deployed at the conscription office in order to identify adolescents belonging to the study; participants were given a folder summarizing previous findings from the study and requesting them to take part in the new follow-up visit. For girls, the same material was distributed to their home addresses. Using the same strategy as in the 15-years follow-up, participants were requested to invite their friends to participate. Participants who refused to visit the clinic at this initial contact were interviewed at home and invited to visit the clinic to have the measurements taken.

Up to April 2012, 164 deaths had been detected. At the age of 18 years, 4563 members were located, of whom 4106 were interviewed. Those who completed the interviews, added to those known to have died, represented 81.3% of the original cohort. Of those located, 127 (2.3%) refused to participate in the study and 330 (7.2%) were considered losses, 196 were found living in other cities and were not interviewed. Of the 4106 individuals interviewed, 50.9% were girls and the mean age was 18.5 years.

The number of eligible participants for each visit of the cohort and the corresponding attrition rates are shown in Table 1. Table 2 describes the characteristics of the individuals located in the 15- and 18-years follow-ups compared with the full cohort. At both visits, follow-up rates were higher among those from intermediate socioeconomic groups and those born with low birthweight. At 18 years only, those who were born at term and those who were not undernourished at age 2 years were more likely to be located.

**Table 1.** Number of eligible subjects and losses to follow-up for each visit of the 1993 Pelotas (Brazil) birth cohort study

Age	Number of eligible subjects	Losses to follow-up*
Perinatal	5265	0.3%
1 month	655	0.9%
3 months	655	1.7%
6 months	1460	3.2%
1 year	1460	6.6%
4 years	1460	12.8%
11 years	5249	12.5%
15 years	5249	14.3%
18 years	5249	18.7%

*Subjects known to have died were considered as traced in all follow-up visits.

**Table 2.** Follow-up rates at 15 and 18y according to baseline characteristics

Variable	Original N (1993)	% interviewed (15y)*	P**	% interviewed (18y)*	P**
Sex	5248		0.049		0.149
Male	2603	78.0		77.4	
Female	2645	82.1		79.1	
Household income (mw)+	5249		<0.001		0.005
≤ 1	967	80.9		75.6	
1.1 to 3.0	2260	81.9		78.1	
3.1 to 6.0	1204	83.6		81.8	
6.1 to 10.0	433	74.6		76.4	
> 10.0	385	75.8		76.1	
Maternal schooling (years)	5246		<0.001		<0.001
0	134	76.1		69.4	
1 to 4	1338	80.9		75.0	
4 to 8	2424	83.5		80.9	
≥ 9	1350	77.2		77.5	
Birthweight (g)	5232		0.008		<0.001
< 2500	510	85.3		72.4	
2,500 to 3,499	3361	79.9		77.8	
≥3500	1361	82.0		81.9	
Gestational age (weeks)	5171		0.093		<0.001
< 37	589	83.5		73.0	
≥ 37	4582	80.6		79.3	
Weight/length (z score)	4947		0.934		0.555
< −2	179	81.6		76.0	
−2 to +2	4572	80.6		79.3	
> +2	196	80.1		79.1	
Length/age (z score)	5118		0.286		0.006
< −2	551	83.3		75.1	
−2 to +2	4509	80.6		79.2	
> +2	58	82.8		91.4	
Weight/age (z score)	5189		0.002		<0.001
< −2	448	86.2		71.2	
−2 to +2	4679	80.4		78.9	
> +2	62	90.3		90.3	
Total	5249	81.1	–	78.2	–

*Those who had died were considered as found (n=163); **Chi-squared test; + Minimum wage.

## What has been measured?

In the 2008 follow-up visit, the mothers or adult caregivers and the adolescents were interviewed. Table 3 describes the main variables collected in this visit according to the four main analytical categories: mental health; body composition; risk factors for NCDs; and human capital. DNA was extracted from saliva using the Oragene® kit and a drop of digital pulp capillary blood was collected on Whatman® cards, one per participant. Genotyping of the interleukin-4 gene polymorphisms (rs2243250 and rs2070874) and methylation analysis of a promoter region of the same gene were also performed. Total IgE levels were analysed from digital pulp blood sample.

**Table 3.** Main categories of variables collected in the most recent (15 and 18 years) follow-up visits. Pelotas 1993 Birth Cohort Study

Visits	Main variables collected	Sample size	
		*Main follow-up visit*	*Sub-studies*
15 years	Mental health: Strengths and Difficulties Questionnaire^15^, stressful events	N= 5249	–
	Body composition: weight, waist and hip circumference, triceps and subscapular skinfolds		
	Risk factors for non-communicable diseases: smoking, alcohol intake, diet, physical activity, sedentary behaviour, violence, blood pressure, lung function, blood collection		
	Human capital: Socioeconomic status, marital status, education, employment, reproductive history, height		
18 years	Mental health: Mini International Neuropsychiatric Interview^16^, stressful events, self-reported questionnaire brief^17^, happiness^18^	N= 5249	Oral health N=1019
	Body composition: dual-energy X-ray absorptiometry (DXA)^19^, plethysmograph (BodPod)^20^, photonic scanner (3- DPS)^21^, weight, waist circumference, triceps and subscapular skinfolds, deuterium, adductor muscle of thumb		Deuterium N=465
	Risk factors for non-communicable diseases: smoking, alcohol intake, diet, objectively-measured physical activity^22^, sedentary behaviour, violence, blood pressure, lung function, carotid ultrasound, blood collection		Adductor muscle of thumb N=465
	Human capital: Socioeconomic status, marital status, education, employment, reproductive history, height, intellectual quotient^23^^,^^24^, quality of life^25^		Qualitative study (obesity) N=80

Table 3 also presents information collected at 18 years of age. DNA was extracted from venous blood collected with EDTA by salting out. Serum, plasma, whole blood and DNA samples are stored at appropriate temperatures. Levels of glucose, cholesterol, triglycerides, HDL-cholesterol, LDL-cholesterol and ultrasensitive C-reactive protein (us CRP)will be measured from serum samples. Glycated haemoglobin levels were evaluated in the last follow-up samples and analyses are ready to use. A subsample of participants also underwent measurements of the thumb adductor muscle strength (*n* = 465), oral cavity examination (*n* = 1019) and saliva collection for deuterium body composition analysis (*n* = 465). The full visit to the clinic including interview and measurements lasted on average 4 h.

The questionnaires and interviewer guides from all follow-up visits are available in electronic and paper formats (http://www.epidemio-ufpel.org.br/site/content/coorte_1993/index.php). In all phases of the study, ethical approval was obtained from the Medical School Ethics Committee of the Federal University of Pelotas and full informed consent was provided by parents (if the subject was aged under 18 years) or by cohort members.

## What has it found? Key findings and publications.

Most of the results presented below have not been published yet (Table 4).

**Table 4.** Comparison of selected results collected at the 15 and 18 year follow up waves of the 1993 birth cohort

Indicators	15 years %	18 years %
Attended school last year	98.0	82.0
Any paid work last year	22.2	88.6
Household income (median in Brazil Reais)	$ 800	$ 1422
Ever had a child	0.5	9.8
BMI for age (<1 SD)	70.9	71.4
Ever had sexual intercourse	19.8	80.0
Wheezing last year	12.1	12.8
Ever had a fracture	19.2*	22.3
Ever smoked	18.8	22.5
Ever consumed alcohol	58.6	91.7
Well-being: very happy in last year (face scale)	38.1	28.3

*Information provided by the mother or caring adult.

### Mental health

The first and second most prevalent mental disorders were agoraphobia (36%) and generalized anxiety disorder (10%). We found that 7% of the cohort members were depressed at 18 years of age. In terms of early-life determinants of adolescent mental health, we found that maternal smoking during pregnancy was related to a higher risk of offspring depression at 18 years of age.^26^ Also a dose-response association was found between number of cigarettes smoked per day during pregnancy and risk of offspring’s depression at 18 years of age.^26^

### Body composition

The prevalence of obesity at 18 years of age was 10% using the BMI–for-age WHOcriterion. Participants had, on average, 17 kg of fat mass (13 kg among boys and 21 kg among girls). The mean bone mineral density of the total body was 1.8 g/cm^2^ [standard deviation (SD) 1.0]. Maternal smoking during pregnancy was negatively associated with offspring bone mass at 18 years, particularly among boys. Also, birthweight was positively associated with bone mass in both sexes. Active commuting throughout adolescence was associated with lower levels of central body fat at 18 years of age in boys but not in girls.

### Risk factors for NCDs

The proportion of 18-year-olds reaching the 300 min/week physical activity recommendation according to self-report was 44%. Physical activity was also assessed through accelerometry in the entire cohort at the age of 18 years. Boys were more active than girls, and the acceleration mean was linear and inversely associated with socioeconomic position. Mean FEV_1_ and FVC were, respectively, 3.6 (SD 0.8) and 4.2 l (SD 0.9). Change in waist circumference from 15 to 18 years and percentage body fat at 18 years old were inversely related to lung function parameters at 18 years.

### Human capital

Happiness was evaluated for the first time in the cohort at the 18 years of age follow-up visit. We found that 32% of the participants were above the threshold for happiness. Maternal and paternal smoking during pregnancy were related to a lower likelihood of offspring's happiness at 18 years of age.^26^ Participants were exposed to more health and social risk factors, and showed elevated rates of conduct problems and violence, compared with children in the Avon Longitudinal Study of Parents and Children (ALSPAC), UK. The prediction of perinatal risk factors for conduct and violence problems was found to be very weak.

## What are the main strengths and weaknesses?

The success of the 2008 and 2011 follow-ups is confirmed by the low percentages of losses and refusals, which minimize the likelihood of bias. Our response rates are comparable to those obtained in other birth cohort studies, such as the 1982 Pelotas cohort, the Birth to Twenty Cohort from South Africa and the Avon Longitudinal Study of Parents and Children (ALSPAC) from England. In addition, the multidisciplinary nature of the cohort was expanded, which required involving professionals from various fields (e.g. biochemistry, anthropology, genetics, physical education) and building a broader view of health.

An important challenge in the most recent visits of the cohort was to conduct all interviews and examinations within a reasonable time frame. Visits at 15 and 18 years of age lasted on average around 3 and 4 h, respectively, requiring long duration of commitment from the participants and the research team.

Participants with worse socioeconomic and nutritional profiles were slightly less likely to be followed up. Socioeconomically intermediate participants were more likely to located as compared with very poor or very rich individuals. Furthermore, participants whose mothers had no schooling were less likely to be followed up. In terms of growth indicators, those with worse nutritional conditions were less likely to participate in follow-up visits. It should be noted, however, that the magnitude of such differences is modest, therefore minimizing the likelihood of bias.

## Can I get hold of the data? Where can I find more?

We welcome requests for data analyses from outside scientists. In our experience, the most rewarding experience is when colleagues from other parts of Brazil or from abroad come to Pelotas to get to know the cohorts and the datasets. We often host such researchers as well as graduate students, who work alongside one or more local researchers to receive training or discuss analyses and possibilities of investigations based on the 1993 birth cohort database. Since 2005, students from Latin America have been accepted in a specific postgraduate programme funded by the Wellcome Trust. These students are involved in our birth cohorts, and use these data for their dissertations. For more information, refer to our website (http://www.epidemio-ufpel.org.projetos_de_pesquisas/estudos/coorte_1993) or e-mail the corresponding author.

## Funding

The 1993 birth cohort study is currently supported by the Wellcome Trust through the programme entitled Major Awards for Latin America on Health Consequences of Population Change. The European Union, National Support Program for Centers of Excellence (PRONEX), the Brazilian National Research Council (CNPq), the Foundation for Research Support of the State of Rio Grande do Sul (FAPERGS) and the Brazilian Ministry of Health supported phases of the study.
